# Suitable DNA Barcoding for Identification and Supervision of *Piper kadsura* in Chinese Medicine Markets

**DOI:** 10.3390/molecules21091221

**Published:** 2016-09-12

**Authors:** Ning Yu, Hong Gu, Yulong Wei, Ning Zhu, Yanli Wang, Haiping Zhang, Yue Zhu, Xin Zhang, Chao Ma, Aidong Sun

**Affiliations:** 1 College of Biological Sciences and Technology, Beijing Forestry University, Beijing 100083, China; yuning56@bjfu.edu.cn (N.Y.); guhongh9@163.com (H.G.); weiyulong1989@126.com (Y.W.); ningxin1127@126.com (N.Z.); wangyanli@bjfu.edu.cn (Y.W.); z624571440@163.com (H.Z.); zhuyue1109@163.com (Y.Z.); someone123667@sohu.com (X.Z.); 2 Beijing Key Laboratory of Forest Food Processing and Safety, Beijing Forestry University, Beijing 100083, China

**Keywords:** *Piper kadsura*, DNA barcoding, *psbA-trnH*, medicine market supervision

## Abstract

*Piper kadsura* is a vine-like medicinal plant which is widely used in clinical treatment. However, *P. kadsura* is often substituted by other materials in the markets, thereby causing health risks. In this study, 38 *P. kadsura* samples and eight sequences from GenBank, including a closely-related species and common adulterants were collected. This study aimed to identify an effective DNA barcode from four popular DNA loci for *P. kadsura* authentication. The success rates of PCR amplification, sequencing, and sequence acquisition of *matK* were 10.5%, 75%, and 7.9%, respectively; for *rbcL* they were 89.5%, 8.8%, and 7.9%, respectively; ITS2 rates were 86.8%, 3.0%, and 2.6%, respectively, while for *psbA-trnH* they were all 100%, which is much higher than for the other three loci. The sequences were aligned using Muscle, genetic distances were computed using MEGA 5.2.2, and barcoding gap was performed using TAXON DNA. Phylogenetic analysis showed that *psbA-trnH* could clearly distinguish *P. kadsura* from its closely related species and the common adulterant. *psbA-trnH* was then used to evaluate the fake proportions of *P. kadsura*. Results showed that 18.4% of *P. kadsura* samples were fake, indicating that adulterant species exist in the Chinese markets. Two-dimensional DNA barcoding imaging of *P. kadsura* was conducted, which was beneficial to the management of *P. kadsura*. We conclude that the *psbA-trnH* region is a powerful tool for *P. kadsura* identification and supervision in the current medicine markets.

## 1. Introduction

*Piper kadsura* (Choisy) Ohwi is a vine-like medicinal plant found mostly in the Fujian, Zhejiang, and Guangdong provinces of China. The stem part of *P. kadsura* is a traditional medicine in China known as “haifengteng”, and is harvested in summer or autumn. According to Chinese medicinal theory, *P. kadsura* is generally used to dredge meridian, expel wind-dampness, and relieve limb pain [1]. It is also used for cooking and improving digestive function in Japan, because its fruit is similar to pepper. To date, various constituents have been isolated from *P. kadsura*, including amides, lignans, terpenes, and cyclohexanes, which possess anti-human hepatitis B virus, anti-platelet activating factor, anti-insect feeding, and anti-inflammatory activities [2,3]. *P. kadsura* has been widely used in medical treatment and has attracted considerable attention because of its many functions.

*P. kadsura* as sold in medicine markets is always dried or sliced, which makes the identification of its traditional morphological characteristics difficult. Given this, it is frequently replaced by other herbs in clinical treatment, causing potential medical problems, thereby eroding consumers’ confidence. Several closely related species and other adulterants of *P. kadsura* are usually adopted as alternative herbs in many situations. *Piper wallichii*—a closely related species of *P. kadsura* that has no anti-inflammatory action—is often used as a substitute for *P. kadsura* in Zhejiang, Fujian, and Hunan provinces. Both species have the same name and are obtained in the same location. *P. kadsura* is commonly substituted by *Kadsura heteroclite* in Guangxi and Guangdong provinces, which exhibits different healing effects. The application of alternatives, to a certain extent, influences the curative effect. In extreme cases, this behavior may lead to major medical accidents. Therefore, the correct identification of *P. kadsura* is especially critical, and a practical and effective method is urgently needed.

DNA barcoding makes use of short but specific DNA tags or “barcodes”—parts of genes present in all living things—to distinguish one species from another [4]. “Barcode” is a term first coined by Hebert [5]. Many DNA barcodes have been searched. Among them are four loci—namely, *matK*, *rbcL*, ITS2, and *psbA-trnH*—which are the most well-known in the identification of medicinal plants. The *matK* gene of 1084 plant species was studied, and it could be used as a standard DNA barcode for flowering plants [6]. Chase et al. concluded that *rbcL* sequence variation is appropriate for phylogenetic analysis at the taxonomic level of seed plants [7]. Chen et al. concluded that ITS2 could be a good DNA barcode for the authentication of medicinal plants and their closely related species. They also demonstrated that *psbA-trnH* is excellent for species identification [8]. Combination with the above loci for use in species authentication has been widely investigated [9,10,11,12,13]. In this study, we utilized DNA barcoding to discriminate *P. kadsura* species from its closely related species and common adulterants. This technique demonstrated that *psbA-trnH* is the best sequence for *P. kadsura* identification.

DNA barcoding has been increasingly used for supervision of the medicine market [14,15,16]. Chen et al. established a barcode system, called traditional Chinese Medicine Database, based on a combination of the ITS2 and *psbA-trnH* barcodes [17]. The system contains 78,847 sequences belonging to 23,262 medicinal species, and covers more than 95% of the herbs in pharmacopeia, including those of China, Japan, Korea, India, the USA, and Europe. The sequences provide consumer protection and safety. In the present study, we used *psbA-trnH* to supervise commercial *P. kadsura* products in the current markets. Results indicated that adulterants are present, and that *psbA-trnH* is a powerful identification tool for *P. kadsura*. Portable equipment based on DNA barcoding technology is desired in the future.

## 2. Results

### 2.1. Success Rates of PCR Amplification, Sequencing, and Sequence Acquisition

**Figure 1:**
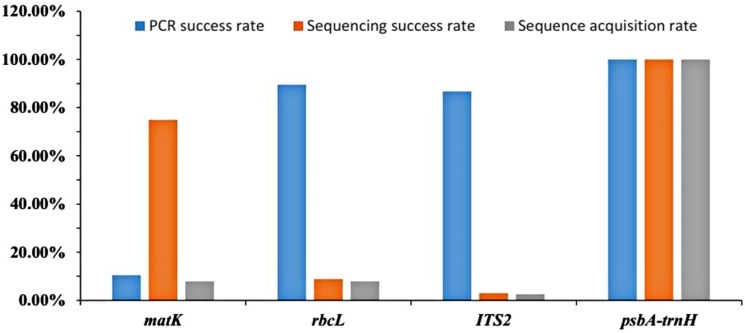
The success rates of PCR amplification, sequencing, and sequence acquisition.

Unsuccessful sequences require repeat experiments or PCR amplification reconstruction to ensure data reliability. The success rate of PCR amplification refers to the rate of samples with evident DNA straps in all the text samples. The success rate of sequencing is the rate of samples with high-quality sequences in all sequences. Sequence acquisition rate is the product of the success rate of PCR amplification and the success rate of sequencing. As shown in Figure 1, all three factors of four loci were investigated. Among the 38 samples, only four samples could be amplified with the *matK* locus. The amplification rates of *rbcL* and ITS2 were 89.5% and 86.8%, respectively; however, the success rates of sequencing were 8.8% and 3.0%, respectively. We attempted to perform repeat experiments for the two loci, but the results remained unsatisfactory. Thus, the sequence acquisition rate of ITS2 was 2.6%; *matK* and *rbcL*, 7.9%; and *psbA-trnH*, 100%. Given that the sequence acquisition rate of *psbA-trnH* was much greater than ITS2, *matK*, and *rbcL*, *psbA-trnH* was chosen as the DNA barcode for *P. kadsura*.

### 2.2. Genetic Distances within and between Species

Basic Local Alignment Search Tool (BLAST) analyses were performed on the 38 sequences. The results indicated that HF007AG07, HF009AG09, HF021YZ01, HF022YZ02, HF023YZ03, HF028YZ08, and HF038BZ05 were not *P. kadsura* samples, but were the same adulterant (i.e., *Actinidia chinensis*), and we treated these samples as AC. The kimura-2-parameter genetic distances of all 46 sequences were calculated. The intraspecific distances of *P. kadsura* was 0.000. The interspecific distance between *P. kadsura* and its adulterants varied from 0.004 to 0.426. The minimum interspecific distance of 0.004 between *P. kadsura* and *Piper wallichii* was larger than the maximum intraspecific distance, proving that *psbA-trnH* is a useful identification tool (Table 1).

### 2.3. Barcoding Gap Assessment

**Figure 2:**
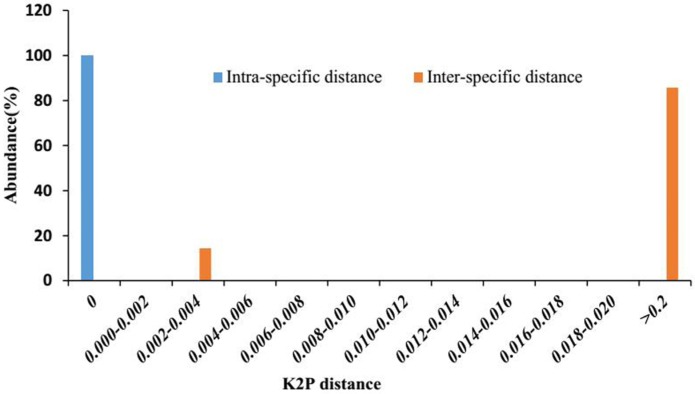
Relative distribution of interspecific divergence between congeneric species and intraspecific distances for *psbA-trnH* locus. K2P: kimura-2-parameter.

Barcoding gap is an important index to determine whether or not a DNA barcode is suitable. Barcodes should exhibit a “barcoding gap” between *intra* and *interspecific* divergences [6]. The divergence distributions were classed in 0.002 distance units to evaluate whether or not the gap exists. With *psbA-trnH* in the *P. kadsura* and its adulterants matrix, there were no overlaps, and the distributions of *intra* and *interspecific* divergence were well separated, forming an evident gap, which is a positive proof for *psbA-trnH* identification ability (Figure 2).

### 2.4. Neighbor-Joining (NJ) Tree Identification

**Figure 3:**
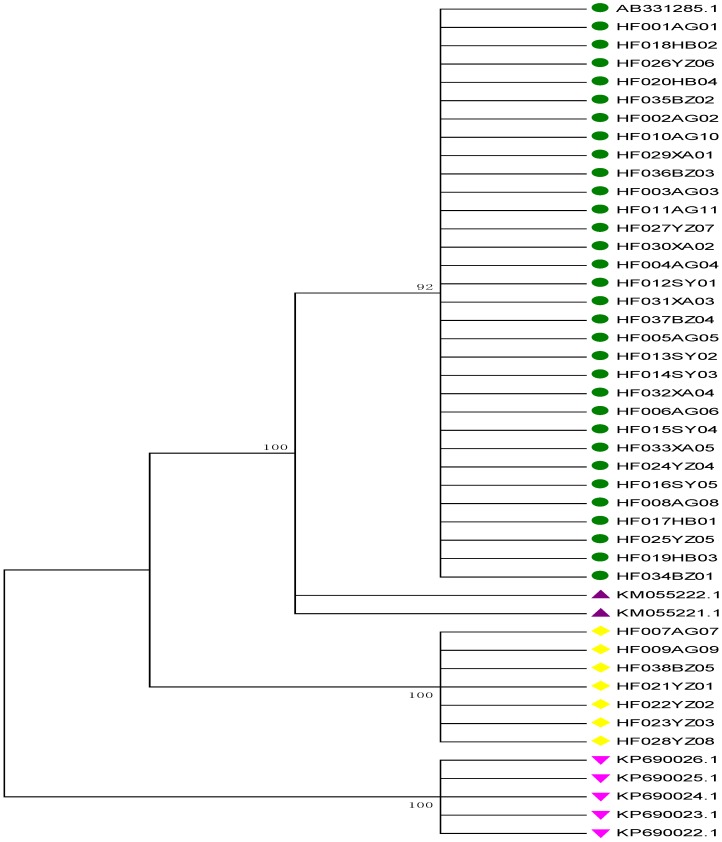
Phylogenetic analysis of *psbA-trnH* regions for *P. kadsura* and its adulterants.

An NJ tree was constructed using 46 *psbA-trnH* sequences (Figure 3). A total of 31 *P. kadsura* sequences clustered into one clade with the *P. kadsura* sequence downloaded from GenBank, whereas its adulterants clustered into other clades. Surprisingly, adulterants were neither *P. wallichii* nor *K. heteroclite*. Seven samples from Hebei Anguo (AG), Henan Yuzhou (YZ), and Anhui Bozhou (BZ) were identified as *Actinidia chinensis* stem. In addition to common adulterants, other fake samples that could not be easily identified by morphology were also present. Thus, DNA barcoding is necessary for authentication. We conclude that the NJ tree could effectively identify *P. kadsura* from its adulterants, including its closely related species *P. wallichii*, which further indicates that *psbA-trnH* is a suitable DNA barcode for *P. kadsura* identification.

### 2.5. Proportions of Adulterant Species

**Figure 4:**
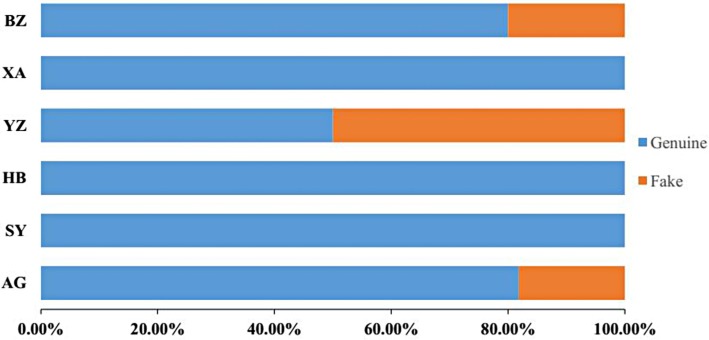
The adulterant rate observed for 38 *P. kadsura* samples.

Product adulteration and ingredient substitution are common as species of a lower market price are used to replace those of a higher price. The frequency of product mislabeling in herbal markets is estimated at 14% to 33% [18]. In this study, 38 *psbA-trnH* sequences were searched to evaluate adulterant proportions of *P. kadsura* in the current markets. The analysis of *psbA-trnH* region identification showed that 18.4% of samples were different from their commercial names. No fake *P. kadsura* samples were found in Hunan Shaoyang (SY), Heilongjiang Haerbin (HB), or Shanxi Xian (XA). The fake sample rates for AG and BZ were 18.2% and 20%, respectively, whereas YZ obtained the highest rate at approximately 50% (Figure 4). The existence of adulterants poses health risks and seriously delays the development of traditional medicine.

### 2.6. DNA Barcoding and Two-Dimensional DNA Barcoding Image

**Figure 5:**
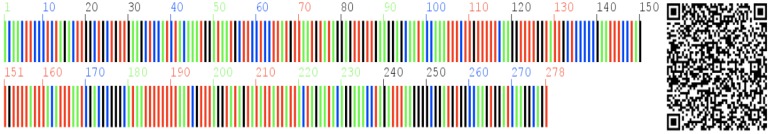
DNA barcoding and two-dimensional DNA barcoding image of *psbA-trnH* sequences for *P. kadsura* (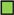
**A**
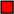
**T**
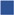
**C**
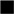
**G**).

DNA barcoding technology aims to identify species, similar to the barcoding of goods in the supermarket. Based on the written code and open-source PHP QR Code coding method [19], the *psbA-trnH* sequences of *P. kadsura* were converted into two-dimensional DNA barcoding images (Figure 5). Two-dimensional DNA barcoding is the combination of two-dimensional technique and DNA barcoding, which is beneficial for the information conversion of DNA barcoding. Different colors represent different nucleotides, and numbers represent the length of the sequence. The sequence of *P. kadsura* can be obtained by scanning the two-dimensional code of the mobile terminal and then sending this sequence to a DNA barcoding database for identification, which makes identification more convenient and quick and facilitates the management of *P. kadsura*. This method can be applied to the identification of other medicinal herbs, to provide a new technical means for the protection of clinical safety.

## 3. Discussion

### 3.1. Efficacy of psbA-trnH for Identification

The non-coding *psbA-trnH* spacer is the most variable plastid region in angiosperm, and it remains the leading candidate for plant DNA barcoding. The *psbA-trnH* spacer satisfies the criteria deemed appropriate for a plant barcode, including short length (often <500 bp) that allows for easy DNA extraction and amplification, high interspecific variability and divergence, and universal flanking primers [20,21,22]. The *psbA-trnH* intergenic spacer region could be used as a barcode to distinguish various *Dendrobium* species and differentiate *Dendrobium* species from other adulterating species [23]. Kress et al. indicated that the rate of PCR success of *psbA-trnH* with standard primers is 95.8% (46 of 48 genera), which is the highest in the nine loci searched [9]. The *psbA-trnH* region ranked first in divergence value in six of the eight genera, and in 11 of the 14 species pairs, compared with the other eight plastid regions [24]. Moreover, *psbA-trnH* alone showed the highest rate of identification (90%), much higher than *rbcLa* and *matK* [25]. Ma et al. concluded that *psbA-trnH* is a powerful DNA marker for medicinal *Pteridophytes* identification [26]. In the present study, the *psbA-trnH* region showed the highest success rates of PCR amplification and sequencing, as well as sequence acquisition rate in *P. kadsura*, which was far higher than the other three loci. The inter-specific divergence of *psbA-trnH* was much greater than intra-specific, creating an evident barcoding gap. In addition, the *psbA-trnH* sequence could clearly identify *P. kadsura* from its adulterants, including closely related species, according to NJ tree description. The two-dimensional DNA barcoding image of *psbA-trnH* sequences for *P. kadsura* also verify the importance of DNA barcoding. Our research demonstrated that *psbA-trnH* is a useful tool for the identification of *P. kadsura*.

### 3.2. Opportunities and Challenges of DNA Barcoding in Medicine Markets

DNA barcoding is a novel system designed to provide rapid, accurate, and automated species identification by using short, standardized gene regions as internal species tags [27]. In recent years, DNA barcoding has played an increasingly significant role in the identification of Chinese medicines. Several studies were conducted to apply DNA barcoding for supervision in medicine markets. Han et al. used barcoding to investigate the proportions and varieties of adulterant species in traditional Chinese medicine (TCM) markets. A total of 1436 samples representing 295 medicinal species from seven primary TCM markets were supervised. The results showed that of the successfully generated samples, approximately 4.2% were adulterants. They suggest that a DNA barcode platform should be established for TCM market investigation [28]. Zhao et al. indicated that the ITS2 barcode could effectively identify *Acanthopanacis* cortex, and DNA barcoding is a convenient tool for medicine market investigation [29]. In the current study, 18.4% of *P. kadsura* samples were not genuine. Uncommon adulterants, such as *A. chinensis*, also existed. Three of the six markets were identified with fake medical materials. Certainly, *psbA-trnH* is a favorable control monitor for *P. kadsura* quality. Thus, the current TCM markets should be regulated through a DNA barcoding method, considering the consumer’s trust and clinical safety.

However, many aspects were considered in the determination of medicine quality. For instance, in the pharmacopoeia of China, medicinal effective ingredients are an important index. Whether the herbs that were identified as genuine using DNA barcoding meet the qualification of medicinal effective ingredients is also a significant topic. In addition, morphological methods still play a fundamental role in medicine identification. Thus, morphological and chemical information is necessary to integrate DNA barcoding and achieve maximum efficiency for medicinal material identification [28].

## 4. Experimental Section

### 4.1. Plant Materials

Many medical materials are dried in medicine markets. To determine the efficacy of DNA barcoding identification, we collected 38 dried *P. kadsura* samples from six approved national herbal medicine markets from six provinces in China, including 11 samples from Hebei Anguo (AG), 5 samples from Hunan Shaoyang (SY), 4 samples from Heilongjiang Haerbin (HB), 8 samples from Henan Yuzhou (YZ), 5 samples from Shanxi Xian (XA), and 5 samples from Anhui Bozhou (BZ) (Table 2), which could reflect *P. kadsura* quality condition in the current market. All 38 specimens were deposited in Beijing Forestry University. One *P. kadsura* sequence, two *P. wallichii* sequences, and five *K. heteroclite* sequences were also downloaded from GenBank (Table 3).

### 4.2. DNA Extraction, Amplification, and Sequencing

The samples were first scraped and then wiped with 75% ethanol to prevent fungal contamination. Total DNA extraction was achieved using a plant genomic DNA kit (Tiangen, Beijing, China), based on the Hexadecyltrimethy Ammonium Bromide (CTAB) approach. The primers used for all four regions and the PCR conditions were as follows: ITS2: S2F, ATGCGATACTTGGTGTGAAT; S3R, GACGCTTCTCCAGACTACAAT; PCR conditions: 94 °C 5 min, 40 cycles at 94 °C 30 s, 56 °C 30 s, 72 °C 45 s, and 72 °C 10 min. *psbA-trnH*: fwd. PA, GTTATGCATGAACGTAATGCTC; rev. TH, CGCGCATGGTGGATTCACAATCC; PCR conditions: 94 °C 5 min, 30 cycles at 94 °C 1 min, 55 °C 1 min, 72 °C 1.5 min, and 72 °C 10 min. *matK*: 390F, CGATCTATTCATTCAATATTTC; 1326R, TCTAGCACACGAAAGTCGAAGT; PCR conditions: 94 °C 5 min, 30 cycles at 94 °C 1 min, 48 °C 30 s, 72 °C 1 min, and 72 °C 10 min. *rbcL*: 1f, ATGTCACCACAAACAGAAAC; 724r, TCGCATGTACCTGCAGTAGC; PCR conditions: 95 °C 2 min, 34 cycles at 94 °C 1 min, 55 °C 30 s, 72 °C 1 min, and 72 °C 10 min [8].

### 4.3. Sequence Alignment and Genetic and Phylogenetic Analyses

CodonCode Aligner 4.2.7 (CodonCode Co., Centerville, MA, USA) was used to trim and assemble raw trace files. BLAST analysis was performed using the nucleotide database at National Center for Biotechnology Information (NCBI). Intra- and inter-species genetic distances were obtained with the kimura-2-parameter (K2P) model of MEGA 5.2.2 [30]. Intra- and inter-species pairwise divergences were calculated as a barcoding gap by using TAXON DNA [31]. A neighbor-joining (NJ) tree based on phylogenetic analysis was constructed, with 1000 replicate bootstrap tests to identify *P. kadsura* via MEGA 5.2.2 [30].

## Figures and Tables

**Table 1:** Genetic distances within and between species.

K2P Genetic Distances	Genetic Distance
Intra-specific distances	0.000
Inter-specific distances with *Piper wallichii*	0.004
Inter-specific distances with AC (*Actinidia chinensis*)	0.318
Inter-specific distances with *Kadsura heteroclita*	0.426

**Table 2:** The list of 38 samples used in this study. AG: Hebei Anguo; BZ: Anhui Bozhou; HB: Heilongjiang Haerbin; SY: Hunan Shaoyang; XA: Shanxi Xian; YZ: Henan Yuzhou.

Sl No.	Latin Name	Place of Collection	Sample No.	Identification Result
1	*P. kadsura*	AG	HF001AG01	Genuine
2	*P. kadsura*	AG	HF002AG02	Genuine
3	*P. kadsura*	AG	HF003AG03	Genuine
4	*P. kadsura*	AG	HF004AG04	Genuine
5	*P. kadsura*	AG	HF005AG05	Genuine
6	*P. kadsura*	AG	HF006AG06	Genuine
7	*P. kadsura*	AG	HF007AG07	Fake
8	*P. kadsura*	AG	HF008AG08	Genuine
9	*P. kadsura*	AG	HF009AG09	Fake
10	*P. kadsura*	AG	HF010AG10	Genuine
11	*P. kadsura*	AG	HF011AG11	Genuine
12	*P. kadsura*	SY	HF012SY01	Genuine
13	*P. kadsura*	SY	HF013SY02	Genuine
14	*P. kadsura*	SY	HF014SY03	Genuine
15	*P. kadsura*	SY	HF015SY04	Genuine
16	*P. kadsura*	SY	HF016SY05	Genuine
17	*P. kadsura*	HB	HF017HB01	Genuine
18	*P. kadsura*	HB	HF018HB02	Genuine
19	*P. kadsura*	HB	HF019HB03	Genuine
20	*P. kadsura*	HB	HF020HB04	Genuine
21	*P. kadsura*	YZ	HF021YZ01	Fake
22	*P. kadsura*	YZ	HF022YZ02	Fake
23	*P. kadsura*	YZ	HF023YZ03	Fake
24	*P. kadsura*	YZ	HF024YZ04	Genuine
25	*P. kadsura*	YZ	HF025YZ05	Genuine
26	*P. kadsura*	YZ	HF026YZ06	Genuine
27	*P. kadsura*	YZ	HF027YZ07	Genuine
28	*P. kadsura*	YZ	HF028YZ08	Fake
29	*P. kadsura*	XA	HF029XA01	Genuine
30	*P. kadsura*	XA	HF030XA02	Genuine
31	*P. kadsura*	XA	HF031XA03	Genuine
32	*P. kadsura*	XA	HF032XA04	Genuine
33	*P. kadsura*	XA	HF033XA05	Genuine
34	*P. kadsura*	BZ	HF034BZ01	Genuine
35	*P. kadsura*	BZ	HF035BZ02	Genuine
36	*P. kadsura*	BZ	HF036BZ03	Genuine
37	*P. kadsura*	BZ	HF037BZ04	Genuine
38	*P. kadsura*	BZ	HF038BZ05	Fake

**Table 3:** The list of accessions downloaded from GenBank.

No.	Latin Name	Genus Name	Genebank No.
1	*P. kadsura*	*Piper* Linn.	AB331285.1
2	*P. kadsura*	*Piper* Linn.	KM055222.1
3	*P. wallichii*	*Piper* Linn.	KM055221.1
4	*K. heteroclita*	*Kadsura* Kaempf. ex Juss.	KP690026.1
5	*K. heteroclita*	*Kadsura* Kaempf. ex Juss.	KP690025.1
6	*K. heteroclita*	*Kadsura* Kaempf. ex Juss.	KP690024.1
7	*K. heteroclita*	*Kadsura* Kaempf. ex Juss.	KP690023.1
8	*K. heteroclita*	*Kadsura* Kaempf. ex Juss.	KP690022.1

## Abbreviations

The following abbreviations are used in this manuscript:

TCM: Traditional Chinese medicine
K2P: Kimura 2-Parameter
NJ: Neighbor-joining
